# Brain Organization of *Apolygus lucorum*: A Hemipteran Species With Prominent Antennal Lobes

**DOI:** 10.3389/fnana.2019.00070

**Published:** 2019-07-17

**Authors:** Gui-Ying Xie, Bai-Wei Ma, Xiao-Lan Liu, Ya-Jun Chang, Wen-Bo Chen, Guo-Ping Li, Hong-Qiang Feng, Yong-Jun Zhang, Bente G. Berg, Xin-Cheng Zhao

**Affiliations:** ^1^Department of Entomology, College of Plant Protection, Henan Agricultural University, Zhengzhou, China; ^2^Institute of Plant Protection, Henan Academy of Agricultural Sciences (HAAS), Zhengzhou, China; ^3^State Key Laboratory for Biology of Plant Disease and Insect Pests, Institute of Plant Protection, Chinese Academy of Agricultural Sciences, Beijing, China; ^4^Department of Psychology, Norwegian University of Science and Technology, Trondheim, Norway

**Keywords:** Apolygus lucorum, brain, neuropil, anatomy, antennal lobe

## Abstract

The anatomical organization of distinct regions in the insect brain often reflects their functions. In the present study, the brain structure of *Apolygus lucorum* was examined by using immunolabeling and three-dimensional reconstruction. The results revealed the location and volume of prominent neuropils, such as the antennal lobes (AL), optic lobes (OL), anterior optic tubercles (AOTU), central body (CB), lateral accessory lobes (LAL), mushroom lobes, and distinct tritocerebral neuropils. As expected, this brain is similar to that of other insects. One exception, however, is that the antennal lobes were found to be the most prominent neuropils. Their size relative to the entire brain is the largest among all insect species studied so far. In contrast, the calyx, a region getting direct input from the antennal lobe, has a smaller size relative to the brain than that of other species. These findings may suggest that olfaction plays an essential role for *A. lucorum*.

## Introduction

Like other brains, the insect brain is the association center for receiving sensory input, processing the information, and generating commands to control the behavior (Chapman, 1998). Behavioral and sensory adaptations to the environments of diverse insect species are often reflected in the brain in the form of volumetric varied neuropil structures (Molina et al., 2009; Mysore et al., 2009; Ott and Rogers, 2010; O’Donnell et al., 2011). Brain anatomies of insects from different taxa have been examined, including cockroaches, locusts, bugs, aphids, beetles, moths, butterflies, flies, bees, ants, and wasps. All of them share similar neural components, but each possesses a unique brain. Characteristics of brain components across various species/taxa reflect their specific ecological niches. For example, diurnal butterflies possess a brain including large optic lobes (OLs) whereas the corresponding visual center in the brain of nocturnal moths is relatively small (Montogomery and Ott, 2015). Furthermore, social insects have evolved complicated mushroom bodies in response to the demand for displaying non-stereotyped behavioral responses (Brandt et al., 2005; Mysore et al., 2009). Besides, the requirement for caste-specific behaviors in bees, ants, and wasps is also mirrored in distinct brain regions (Molina et al., 2009; Mysore et al., 2009; O’Donnell et al., 2011).

The mirid bug, *Apolygus lucorum* (Meyer-Dür; Hemiptera: Miridae), established itself as an important pest in China during the recent decade (Lu et al., 2010). The wide behavioral reservoir of *A. lucorum*, including polyphagy, high motility, diapause, dispersal, and migration may facilitate its ecological success and population growth (Lu et al., 2010; Lu and Wu, 2012). What characterizes the neural arrangements underlying such a flexible behavior? Investigating the overall brain anatomy of *A. lucorum* and identifying central neuropil components are the first step in addressing this question. Moreover, Miridae is the largest family of Hemiptera, containing approximately 11,000 species worldwide (Cassis and Schuh, 2012). In addition to *A. lucorum*, many mirid bugs are economically important insects, either as pests of crops or as predators used as biological control agents. So far, however, the anatomical brain structures of this big insect group have not been studied.

In the present study, we examined the brain composition of *A. lucorum* by performing immunolabeling and three-dimensional (3D) reconstruction. These approaches have been successfully used in many other insect species, i.e., the fruit fly, *Drosophila melanogaster* (Rein et al., 2002; Ito et al., 2014); the honeybee, *Apis mellifera* (Brandt et al., 2005); the desert locust, *Schistocerca gregaria* (Kurylas et al., 2008); the heliothine moths, *Heliothis virescens* (Kvello et al., 2009) and *Helicoverpa assulta* (Chen et al., 2016); the hawk moth, *Manduca sexta* (El Jundi et al., 2009); cockroach, *Leucophaea maderae* (Wei et al., 2010); the red flour beetle, *Tribolium castaneum* (Dreyer et al., 2010); the nymphalid butterflies, *Danaus plexippus* (Heinze and Reppert, 2012), *Godyris zavaleta* (Montogomery and Ott, 2015), *Heliconius hecale*, and *Heliconius erato* (Montogomery et al., 2016); the tramp ant *Cardiocondyla obscurior* (Bressan et al., 2015); and the dung beetles, *Scarabaeus lamarcki* and *Scarabaeus satyrus* (Immonen et al., 2017). Similar experimental approaches as used in the abovementioned studies allow one to make comparisons of distinct brain structures across the different species/taxa, including shape, location, composition, and volume. As expected, *A. lucorum* has brain components corresponding with those of other insect species. However, to our surprise, it possesses the biggest antennal lobes (AL) relative to the total brain size among all species studied so far.

## Materials and Methods

### Animals

The experiments were performed on male and female adults of *A. lucorum*. The conditions and procedures for rearing *A. lucorum* in the laboratory have been described previously (Xie et al., 2016b,c). The insects were fed a green bean pod (*Phaseolus vulgaris*) in aerated plastic boxes under the following conditions: 28 ± 1°C, 60% relative humidity, and a 16 h/8 h illumination regime.

### Wholemount Labeling of Brain

In order to examine the neuropil structures of the *A. lucorum* brain, immunolabeling with anti-synapsin antibody was performed. The brain was dissected out in Ringer’s saline [in mM, 150 NaCl, 3 CaCl_2_, 3 KCl, 25 sucrose, and 10 N-Tris(hydroxymethyl)-methyl-2-amino-ethanesulfonic acid, pH 6.9] on ice and then transferred into 4% paraformaldehyde in 0.1 M phosphate-buffered saline (PBS, pH 7.4) to be fixed overnight at 4°C in a refrigerator. Preincubation of the brain with 5% NGS (Sigma, St. Louis, MO, USA) in 0.1 M PBS containing 0.5% Triton X-100 (PBST; 0.1 M, pH 7.4) was performed overnight at 4°C after being rinsed in PBS (4 × 15 min). Then, incubation with the primary antibody, SYNORF1 (Developmental Studies Hybridoma Bank, University of Iowa), at a concentration of 1:100 (with 5% NGS in PBST), was applied at 4°C. After 5 days, the brain was rinsed in PBS, 6 × 20 min. Then, incubation with the secondary antibody, Cy2-conjugated anti-mouse (Invitrogen, Eugene, OR, USA; dilution 1:300 with 1% NGS in PBST), was performed for 3 days at 4°C. Afterward, the brain was washed in PBS, 6 × 20 min, and dehydrated with an ascending ethanol series. Finally, the brain was cleared in methylsalicylate, before being mounted in Permount in a perforated aluminum slide with two glass coverslips.

### Anterograde Staining of the Antennal Lobe Tracts

In order to examine the projection pathway of the antennal lobe tracts, anterograde mass staining experiments with the fluorescent dye Micro-Ruby (tetramethylrhodamine dextran with biotin, Micro-Ruby, Molecular Probes; Invitrogen, Eugene, OR, USA) were performed. The animal was kept in a plastic tube, and the head cuticle was opened to allow access to the antennal lobe. Crystals of the dye were then placed into the antennal lobe by using a needle. To make sure that the dye was absorbed by the neurons, the antennal lobe was slightly damaged with the needle. Afterward, the head was covered with Vaseline and the animal was kept in a refrigerator overnight, at 4°C with a moist filter paper, allowing transportation of the dye in the axons. Then, the brain was dissected out from the head capsule in Ringer’s saline, before being exposed for synapsin immunostaining as described above.

### Confocal Image Acquisition, Neuropil Identification, and Three-Dimensional Reconstructions

All image stacks were acquired by using a confocal laser scanning microscope (LSM 780, META Zeiss, Jena, Germany) with a 10× objective (Plan-Neofluar 10×/0.3) on the anti-synapsin immunolabeled whole-mount preparations. An argon laser, 488 nm line, was used to excite the Cy2, and a HeNe1 laser, 543 nm line, was used for the Micro-Ruby. The resolution of the confocal images was set to 1,024 × 1,024 voxels and the section interval was set to 3 or 4 μm.

The Amira software (AMIRA 5.3, Visage Imaging, Fürth, Germany) was used to create a three-dimensional reconstruction of each identified brain neuropil (Xie et al., 2016b,c). The most prominent and easily recognizable neuropils include the optic lobes (OL), antennal lobes (AL), antennal mechanosensory and motor centers (AMMC), mushroom bodies (MB), central body (CB), protocerebral bridge (PB), anterior optic tubercles (AOTU), lateral accessory lobes (LAL), posterior optic lobes (POTU), gnathal ganglion (GNG), and tritocerebrum (TR). The volumes of all reconstructed brain neuropils were calculated by using the tool of “TissueStatistics” embedded in Amira.

### Statistical Analysis

The volume data of *A. lucorum* brain neuropils of six males and four to five females were subject to statistical analysis. The data of mean, standard deviation, and ratios were calculated by using Excel, and the Mann–Whitney *U* tests were performed by using SPSS (IBM SPSS statistics version 21). In order to make a comparison for corresponding neuropils across insect species with different body sizes, the volumes were normalized by calculating the relative size of the relevant neuropils to the volume of the whole brain.

## Results

### Brain Composition of *A. lucorum*

**Figure 1:**
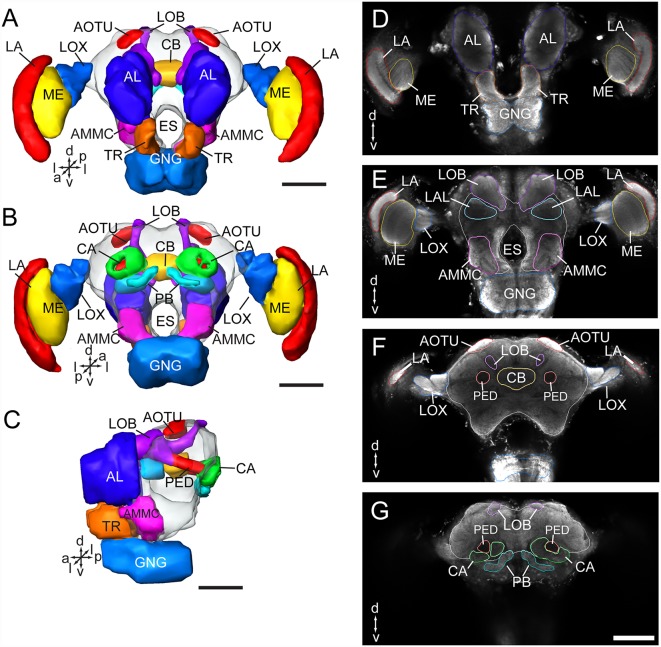
Three-dimensional reconstructions and confocal images of the *Apolygus lucorum* brain. **(A)** Three-dimensional reconstructions of brain in frontal view. **(B)** Posterior view. **(C)** Lateral view. **(D–G)** Serial confocal images showing the brain sections of *A. lucorum*. AMMC, antennal mechanosensory and motor center; AL, antennal lobe; AOTU, anterior optic tubercle; CA, calyx; CB, central body; ES, esophagus; GNG, gnathal ganglion; LA, lamina; LOB, mushroom body lobes; LOX, lobula complex; ME, medulla; PB, protocerebral bridge; PED, pedunculus; TR, tritocerebrum. Directions: a, anterior; d, dorsal; l, lateral; m, medial; p, posterior, v, ventral. Scale bars: 100 μm.

**Figure 2:**
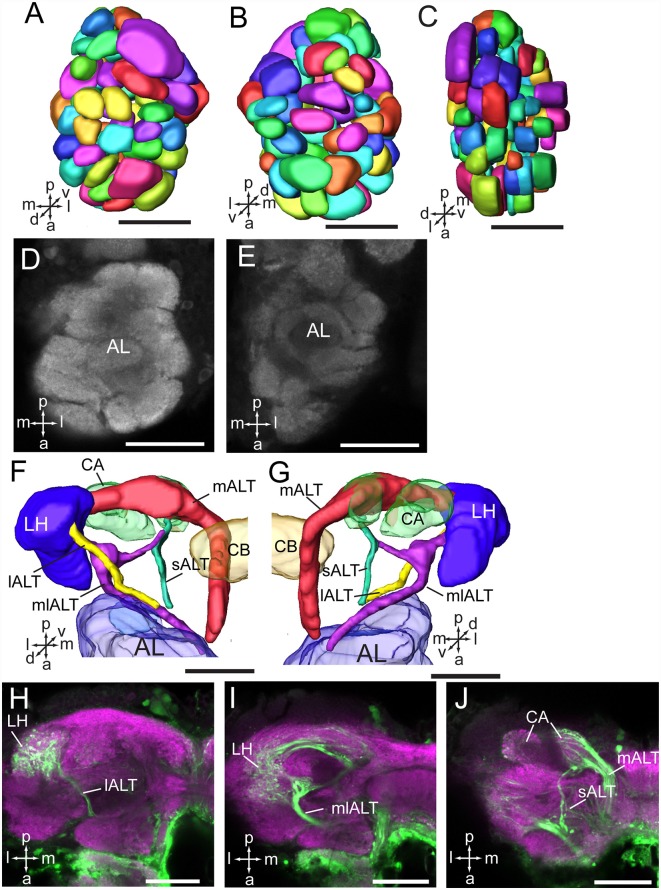
Three-dimensional reconstructions and confocal images of the *Apolygus lucorum* antennal lobe and antennal lobe tracts. **(A)** Three-dimensional reconstructions of antennal lobe glomerulus in dorsal view. **(B)** Vental view. **(C)** Lateral view. **(D,E)** Confocal images of antennal lobe glomeruli at different depths. **(F,G)** Three-dimensional reconstructions of antennal lobe tracts in dorsal view and ventral respectively. **(H–J)** Confocal images of antennal lobe tracts and targeted regions at different depths. AL, antennal lobe; CA, calyx; CB, central body; ES, esophagus; GNG, gnathal ganglion; lALT, lateral antennal lobe tract; LH, lateral horn; mALT, medial antennal lobe tract; mlALT, medial lateral antennal lobe tract; sALT, small antennal lobe tract. Directions: a, anterior; d, dorsal; l, lateral; m, medial; p, posterior, v, ventral. Scale bars: 50 μm.

As in fruit fly, moths, bees, and some other insect species, the supraesophageal and subesophageal ganglia of *A. lucorum* are highly fused and form a brain with a hollow in the middle (Figure 1). The anti-synapsin immunolabeling shows the brain of *A. lucorum* containing the protocerebrum (PR), deutocerebrum (DE), tritocerebrum, and GNG (Xie et al., 2016b). In the PR of *A. lucorum*, the most prominent neuropils, easily identified with the distinct boundaries to the adjacent regions, include the optic lobes, AOTU, CB, protocerebral bridges, pedunculus (PED), calyx (CA), and mushroom body lobes (LOB; Figure 1). In the deutocerebrum, the major neuropils identified were the antennal lobes and the AMMC (Figure 1). The tritocerebrum and GNG, on the other hand, were defined as single neuropils, based on the anti-synapsin immunolabeling (Figure 1). The antennal lobe is composed of mainly spherical glomeruli (Figures 2A–E). There are about 80 glomeruli in each antennal lobe. Tracing experiments revealed that the antennal lobe is linked to the calyx and the lateral horn (LH) *via* the medial, mediolateral, and lateral antennal lobe tracts (lALT; Figures 2F–J). In addition, there is a small tract projecting from the lALT midway between the antennal lobe and the lateral horn, terminating in the calyx (Figures 2F,G,J). The terminals of these tracts were used to determine the regions of the calyx and the lateral horn.

**Figure 3:**
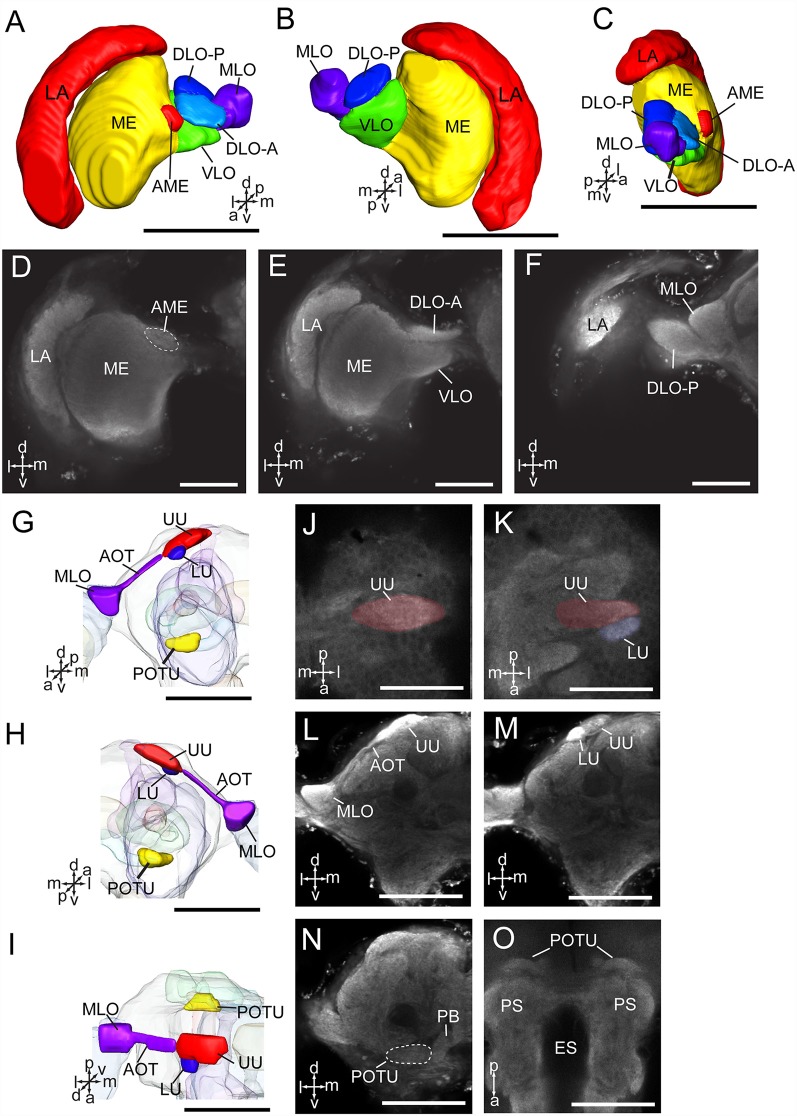
Three-dimensional reconstructions and confocal images of *Apolygus lucorum* optic lobe and optic tubercles. **(A)** Three-dimensional reconstructions of optic lobe in frontal view. **(B)** Posterior view. **(C)** Sagittal view. **(D–F)** Confocal images of optic lobe at different depths. **(G)** Three-dimensional reconstructions of anterior and posterior optic tubercles in frontal view. **(H)** Posterior view. **(I)** Dorsal view. **(J,K)** Confocal images of AOTU in dorsal view. **(L,M)** Confocal images of AOTU in frontal view. **(N)** A confocal image of posterior optic tubercle in frontal view. **(O)** A confocal image of posterior optic tubercle in dorsal view. AME, accessory medulla; AOT, anterior optic tract; DLO-A, anterior part of dorsal lobula; DLO-P, posterior part of dorsal lobula; ES, esophagus; LA, lamina; LU, lower unit of anterior optic tubercle; ME, medulla; MLO, medial lobula; PB, protocerebral bridge; POTU, posterior optic tubercle; PS, posterior slope; UU, upper unit of anterior optic tubercle; VLO, ventral lobula. Directions: a, anterior; d, dorsal; l, lateral; m, medial; p, posterior; v, ventral. Scale bars: 100 μm in **(A–C,G–I,L–O)**, 50 μm in **(D–F,J,K)**.

The optic lobe contains three main neuropils, i.e., the lamina (LA), medulla (ME), and lobula complex (LOX), from distal to medial. The lobula complex is further divided into four lobes, i.e., anterior part of dorsal lobula (DLO-A), posterior part of dorsal lobula (DLO-P), ventral lobula (VLO), and medial lobula (MLO; Figures 3A–F). From the MLO, there are some neurons linking to the AOTU through the anterior optic tract (AOT). The AOTU consists of two parts, an upper unit (UU) and a lower unit (LU; Figures 3G–O). An accessory medulla (AME) was also found in *A. lucorum*, located anteriorly and medially of the medulla (Figures 3A,C,D). In addition, the posterior optic tubercle (POTU) is just posterior to dorsal part of posterior slope, lateral to the lateral end of PB (Figures 3G–I,N,O).

**Figure 4:**
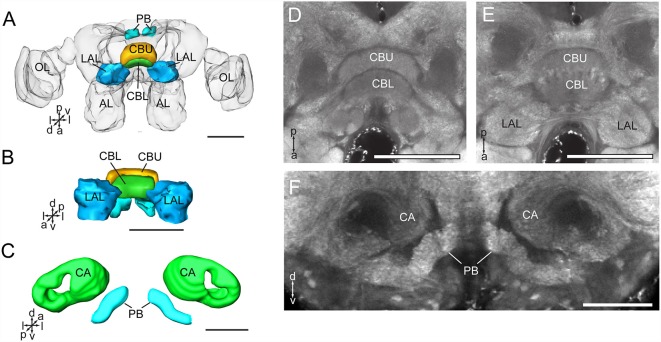
Three-dimensional reconstructions and confocal images of *Apolygus lucorum* CB, PB, and LAL. **(A)** Three-dimensional reconstructions of CB, PB, and LAL in dorsal view. **(B)** Anterior view. **(C)** Three-dimensional reconstructions of PB and calyces in posterior view. **(D)** A confocal image showing the CB. **(E)** A confocal image showing the CB and LAL. **(F)** A confocal image showing the PB and calyces. AL, antennal lobe; CA, calyx; CBL, lower unit of central body; CBU, upper unit of central body; LAL, lateral accessory lobe; OL, optic lobe; PB, protocerebrum bridge. Directions: a, anterior; d, dorsal; l, lateral; p, posterior; v, ventral. Scale bars: 100 μm in **(A,B,D,E)**, 50 μm in **(C,F)**.

The CB is composed of two units, the upper unit (CBU) and lower unit (CBL; Figures 4A,B,D,E). The PB is formed by two separated bar-like structures, located posteriorly to the CB and ventro-medially to each calyx (Figures 4C,F). The CB and the PB form the central complex (CX). No noduli was found in the anti-synapsin immunostained brains of *A. lucorum*. The LAL is located antero-laterally of the CB and posteriorly of the antennal lobe (Figures 2A,B,E).

**Figure 5:**
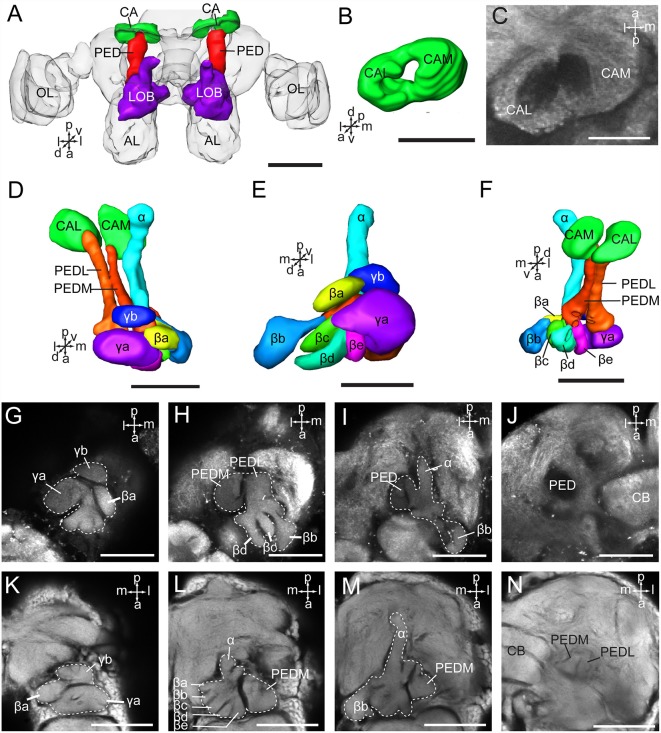
Three-dimensional reconstructions and confocal images of *Apolygus lucorum* mushroom body. **(A)** Three-dimensional reconstructions of mushroom body in dorsal view. **(B)** Three-dimensional reconstruction of calyx in anterior view. **(C)** A confocal image showing the calyx. **(D)** Three-dimensional reconstructions of mushroom body in dorsal view. **(E)** Frontal view. **(F)** ventral view. **(G–J)** Confocal images at different depths showing mushroom body lobes of one preparation. **(K–N)** Confocal images at different depths showing mushroom body lobes of the other preparation. AL, antennal lobe; CA, calyx; CAL, lateral calyx; CAM, medial calyx; CB, central body; PED, pedunculus; PEDL, lateral pedunculus; PEDM, medial pedunculus; α, α lobe; β, β lobe; βa–βe, a–e five β lobelets; γ, γ lobe; γa and γb, a and b two γ lobelets. Scale bars: 100 μm in **(A)**, 50 μm in **(B–N)**.

The mushroom body of *A. lucorum* contains the calyx, pedunculus, and lobes (Figure 5A). It is clear that the calyx is divided into two parts, the lateral calyx (CAL) and medial calyx (CAM), each having the shape of a half cup (Figures 5B,C). The pedunculus is divided into two bundles, the lateral PED (PEDL) and medial PED (PEDM), linking to CAL and CAM, respectively (Figures 5D–N). The lobes are divided in the vertical lobe (α lobe), medial lobe (β lobe), and two bulb-like γ lobes. The medial lobe is further divided into five lobelets, βa–βe (Figures 5E,H,L). The α and β lobes are connected with PEDM, whereas the two γ lobelets, γa and γb, are connected with the PEDL. Mass staining of antennal lobe tracts indicates that the calyx receives direct input from the antennal lobe *via* at least two tracts, the medial and medio-lateral. The medial cup, CAM, displayed extensive staining (Figure 2J).

### Volumetric Comparison of Corresponding Brain Structures in *A. lucorum* and Other Insect Species

**Figure 6:**
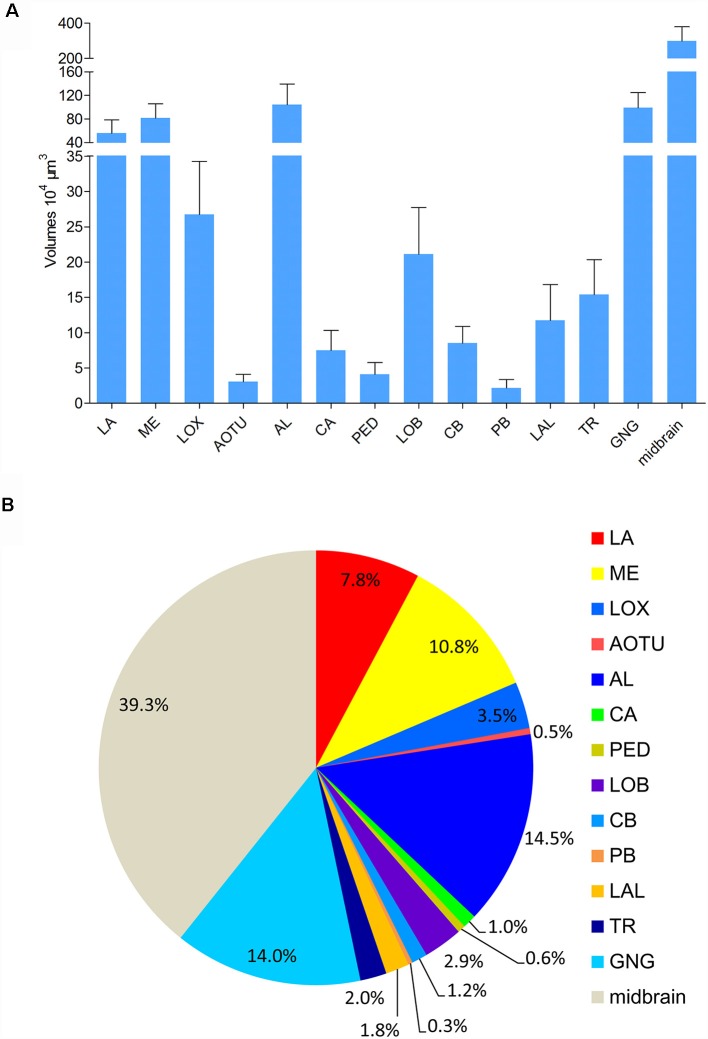
Absolute volumes and relative sizes of prominent neuropils in the brain of *Apolygus lucorum*. **(A)** Absolute volumes. **(B)** Relative size (i.e., size relative to the volume of the whole brain). AL, antennal lobe; AOTU, anterior optic tubercle; CA, calyx; CB, central body; GNG, gnathal ganglion; LA, lamina; LAL, lateral accessory lobe; LOB, mushroom body lobes; LOX, lobula complex; ME, medulla; PB, protocerebral bridge; PED, pedunculus; TR, tritocereburm. Midbrain, the remaining neuropils in the central brain except MB, CB, PB, LAL, and AOTU.

Absolute volumes of relevant brain structures, i.e., prominent neuropils displaying distinct boundaries, were calculated by using the tool of AMIRA (Figure 6; Supplementary Table S1). No differences were found in volumes of bilateral neuropils in the right and left hemisphere, respectively. In addition, no volume differences were found in corresponding neuropils in males and females (Supplementary Table S2). The midbrain, including the neuropils in the PR except for the optic lobes, the AOTU, the mushroom bodies, the CB, the PR bridges, and the LAL, covers the major part of the brain (Figure 6). The antennal lobes are the largest neuropil among the prominent neuropils in the brain of *A. lucorum* accounting for 14.5% of the absolute volume.

**Figure 7:**
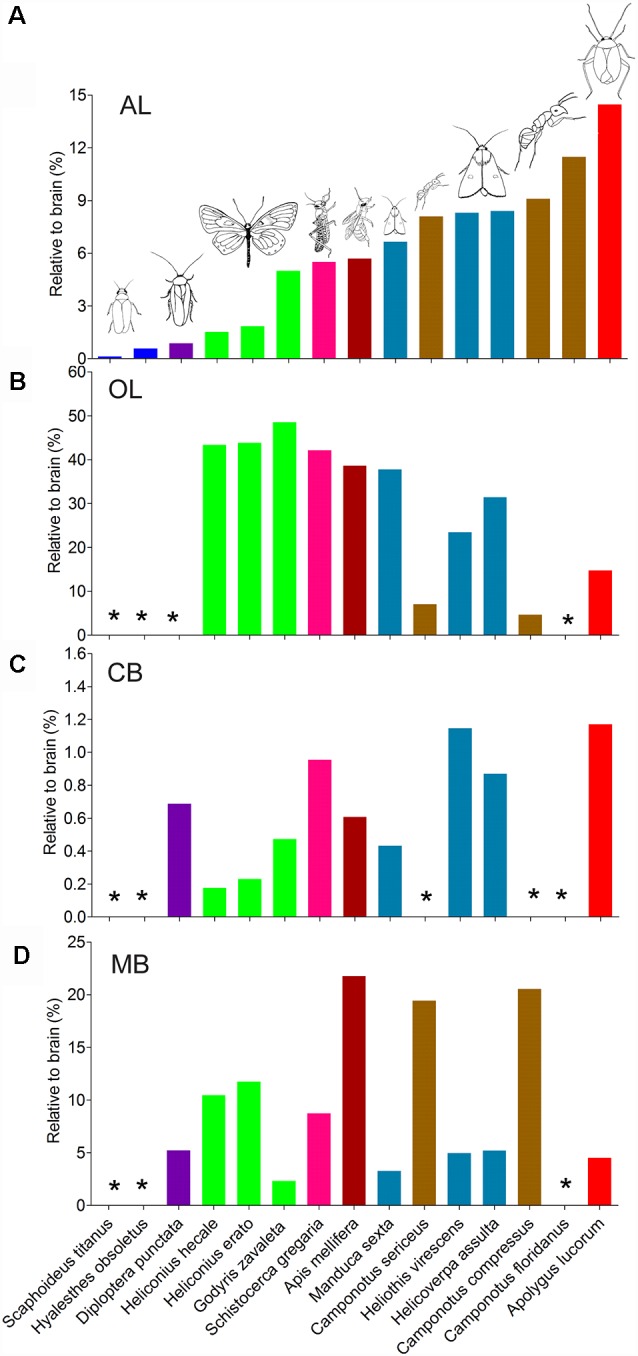
Comparison of relative sizes of the antennal lobe, optic lobe, CB, and mushroom body to the whole brain in different species. **(A)** Antennal lobe. **(B)** Optic lobe including medulla and lobula complex. **(C)** CB. **(D)** Mushroom body. AL, antennal lobe; CB, central body; MB, mushroom body; OL, optic lobe. *Data missing.

The antennal lobes, the optic lobes, the CB, and the mushroom bodies are the most intensively explored neuropils across the different species studied. Similar approaches of immunostaining and 3D reconstruction allow making comparisons of the data obtained here with corresponding findings reported among other species (Figure 7; Supplementary Table S3). In order to compare volumes of corresponding neuropils across different insect species, neuropil size was normalized by relating the real size to the size of the whole brain. Overall, it seems that the relative sizes of corresponding neuropils are quite similar within distinct insect taxa. Calculation of relevant data on the mirid bug, obtained in this study, demonstrates that among all insects investigated, it possesses the relatively largest antennal lobes and CB, while the optic lobes and mushroom bodies are relatively small (Figure 7).

**Figure 8:**
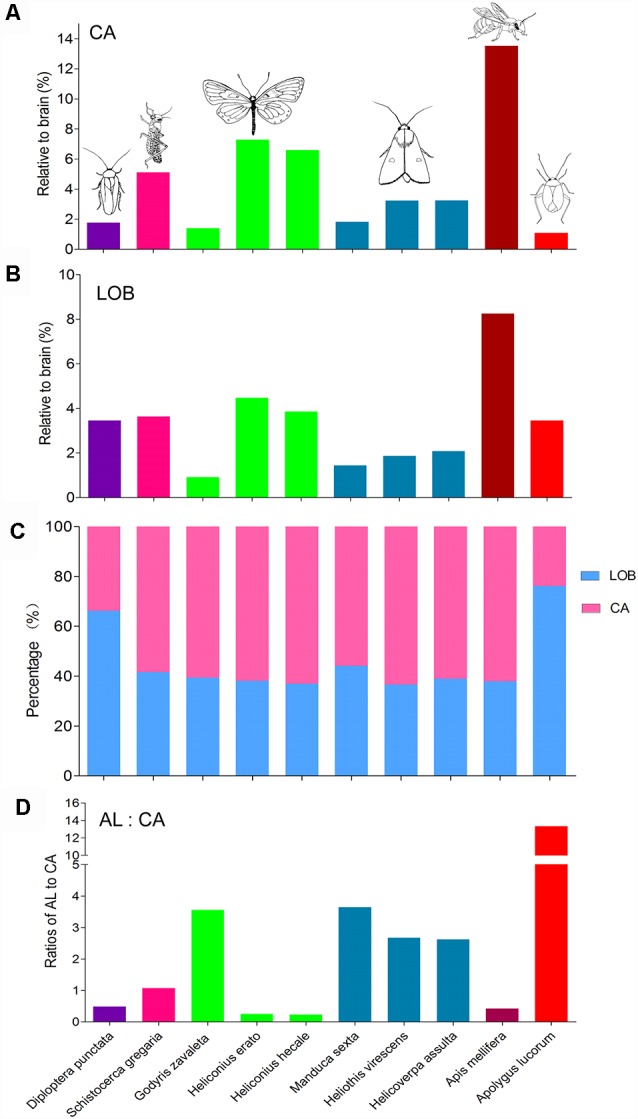
Comparison of relative sizes of the calyx and lobes in different species. **(A)** Relative sizes of the calyx to the whole brain in different species. **(B)** Relative sizes of the mushroom body lobes to the whole brain in different species. **(C)** Proportion of calyx and lobes to the whole mushroom body. **(D)** The ratios of antennal lobe to calyx in different species. AL, antennal lobe; CA, calyx; LOB, mushroom body lobes including the pedunculus.

Besides, the volume of the calyx in *A. lucorum* seems small. Therefore, the relative sizes of the calyx and the mushroom body lobes (here including pedunculus) were also calculated and compared across different species (Figures 8A,B). Among the studied species, the relative size of the calyx is smallest in *A. lucorum*. Here, the calyx constitutes only 24% of the whole mushroom body, while the lobes pose 76%. This is quite different from the arrangement in the desert locust, honeybee, and lepidopteran insects where the proportions of the calyx and the lobes are about 60% and 40%, respectively (Figure 8C). The calyx is innervated by the antennal-lobe projection neurons and it is generally assumed that the volume of the calyx is positively correlated to the antennal lobe volume (Strausfeld et al., 2009). It is obvious that the volume ratio of the antennal lobe to the calyx is far greater in the mirid bug than in the other species (Figure 8D). Surprisingly, in *A. lucorum*, the antennal lobe is large while the calyx is tiny.

## Discussion

Based on successful immunolabeling with anti-synapsin, the brain anatomy of *A. lucorum* was characterized. As expected, the central neuropil structures identified here, including the antennal lobes, optic lobes, CB, PBs, AOTU, mushroom bodies, LAL, and the POTUs, are similar to those previously described in most insects.

### Tritocerebrum and Gnathal Ganglion

The tritocerebrum of *A. lucorum* was identified as a distinct neuropil with obvious boundaries to the adjacent neuropils, like in the previous anatomical study of the desert locust (Kurylas et al., 2008). In flies, moths, and bees, on the other hand, the tritocerebrum is largely reduced and hard to delineate from the surrounding neuropils (Ito et al., 2014). The function of the tritocerebrum is still unclear. However, it is assumed to serve as the primary processing center for taste input from the internal mouthparts (Rajashekhar and Singh, 1994).

Overall, the GNG of *A. lucorum* is fused with the brain, meaning that the supraesophageal and subesophageal ganglia are merged. This arrangement is similar to that found in flies, bees, and moths, but different from that of locusts and cockroaches. Whether the brain is fused with the subesophageal ganglion or not may depend on the diet habits of the insect species rather than on the mode of metamorphism, i.e., whether it is hemi- or holo-metabolous. Actually, insects feeding on solids, such as cockroaches, locusts, beetles, and Lepidoptera larvae, possess a brain that is separated from the GNG (Ito et al., 2014; Tang et al., 2014; Xie et al., 2016a; Immonen et al., 2017). Those feeding on liquids or small particles, on the other hand, such as flies, aphid, mosquitoes, bugs, moths, butterflies, bees, and ants, have a brain that is fused with the GNG (Ignell et al., 2005; Kollmann et al., 2011; Ito et al., 2014; Bressan et al., 2015). Further studies should be performed to test the hypotheses on the above-mentioned relationship of the brain/GNG arrangement with the diet habits.

### Antennal Lobe and Antennal Mechanosensory and Motor Center

Like in other insects, the antennal lobe and the AMMC are two adjacent deutocerebral neuropils of *A. lucorum*, receiving axonal terminals of antennal sensory neurons (Xie et al., 2016c). The AMMC is the primary center for processing information about vibration sound, gravitational force, wind direction, and stability during flight (Sane et al., 2007; Kamikouchi et al., 2009; Yorozu et al., 2009). It receives axonal terminals originating mainly from the neurons of Böhm bristles and Johnston’s organ. Different sensory neurons project to distinct regions of the AMMC in many different insect species (Ma et al., 2017). In *Drosophila*, the AMMC is subdivided into five zones, while in the desert locust, it is subdivided into four regions (Kamikouchi et al., 2009; von Hadeln et al., 2018). In *A. lucorum*, the terminals innervating the AMMC are reported to originate from several different antennal sensory pathways, which may suggest that it consists of several sub-regions as well (Xie et al., 2016c).

The antennal lobe, which is the primary processing center for olfactory information, generally contains many spherical glomeruli (Anton and Homberg, 1999). The number of glomeruli varies from species to species of different taxa, for example, about 50 in each antennal lobe in fruit fly, 60–80 in moths, 160 in honeybee, and 200–600 in ants (Schachtner et al., 2005; Zhao et al., 2016). Each antennal lobe of *A. lucorum* contains about 80 glomeruli (Xie et al., 2016c). In hemipteran species, the number of glomeruli varies extensively. For instance, there are 120–140 glomeruli in the planthopper, *Hyalesthes obsoletus*; 60–80 glomeruli in the stink bug, *Eushchistus heros*; 22 in the blood-sucking bug, *Rhodnius prolixus*; 25–40 in the pea aphid, *Acyrthosiphon pisum*; and 13 in male leafhopper, *Scaphoideus titanus* (Kristoffersen et al., 2008; Barrozo et al., 2009; Kollmann et al., 2011; Rossi Stacconi et al., 2014). Furthermore, in some aphids, psyllid *Trioza apicalis*, and females of *Scaphoideus titanus*, no evident glomeruli exist in the antennal lobe (Kristoffersen et al., 2008; Rossi Stacconi et al., 2014).

### Optic lobe and Anterior and Posterior Optic Tubercle

The optic lobe is the center for processing visual information in the brain, consisting of several neuropils commonly including the lamina, the medulla, the lobula complex, and the AME (Ito et al., 2014). The AME is involved in circadian rhythm and polarized skylight perception (Wei et al., 2010; Beetz et al., 2015). All these neuropils are present in *A. lucorum*. The lamina is most peripherally in the optic lobe, just below the retina. The next neuropil sheet, the medulla, is divided into several layers, always including the outer and inner medulla, and serpentine. In *A. lucorum*, however, the layers of the medulla are not prominent based on the immunostaining with the antibody of synapsin. The third neuropil region, the lobula complex, is reported to display considerable variation. In flies, moths, butterflies, and mosquitoes, the lobula complex is composed of the lobula and the lobula plate. In honeybee, the lobula complex only consists of a single neuropil of the lobula. Furthermore, in locust, mantis, and cockroach, it is divided into three to five sub-regions. The lobula complex of *A. lucorum* is composed of four lobes, similarly to that of the cockroach, locust, and mantis (Rosner et al., 2017), suggesting that the optic lobe of *A. lucorum* is involved in relatively complicated visual functions as well.

The AOTU is a higher-order neuropil for processing visual information including polarized skylight, and it consists of a large UU and a small LU. The LU is divided into a collection of several sub-units in various species, for instance, two in honeybee, three in the monarch butterfly, five in the oriental tobacco budworm *H. assulta*, and six in the two dung beetles (Heinze and Reppert, 2012; Chen et al., 2016; Immonen et al., 2017). In *A. lucorum*, however, the AOTU is composed of a single large UU and a single small LU. These two units are linked to the MLO *via* the AOT.

The POTU is an optic glomerulus identified in *A. lucorum*, positioned laterally to the PB and posteriorly of the posterior slope. The neuropil has previously been described in the desert locust and the monarch butterfly (Heinze and Reppert, 2012; Beetz et al., 2015). In the desert locust, the POTU is connected with the AME, the PB, and the contralateral POTU (Beetz et al., 2015). It is suggested to be involved in a neuronal circuit stabilizing the internal sky compass in the central complex of the locust (Beetz et al., 2015).

### Central Complex and Lateral Accessory Lobe

The central complex is involved in navigation, locomotion, and visual memory (Pfeiffer and Homberg, 2014). This centrally located neuropil consists of four neuropils, including the upper and lower division of the CB, the PB, and the noduli. The organization of the upper and lower divisions of the CB in *A. lucorum* is similar to that of other species, e.g., the desert locust, dung beetles, moths, butterflies, and the fruit fly. The PBs in* A. lucorum* are paired bar-shaped neuropils, similar to those of butterflies and moths (Chen et al., 2016). In *A. lucorum*, however, no noduli was observed.

The LAL is a neuropil that is highly associated with the central complex, mediating the communication between the central complex and the motor centers (Namiki and Kanzaki, 2016). The LAL is present in all studied insect species (Namiki and Kanzaki, 2016). The LAL is subdivided into two or three sub-regions exhibiting different immunoreactivity to antiserum of serotonin and GABA (Namiki and Kanzaki, 2016). The sub-regions of the LAL in *A. lucorum* were not determined based on the immunostaining with anti-synapsin.

### Mushroom Body

The mushroom body serves as a multimodal integration center for learning and memory (Fahrbach, 2006). It is composed of the calyx, pedunculus, and lobes, including vertical and medial lobes. Each component shows considerable variation among different insect species. For instance, the calyx in bees and ants consists of two cup-shaped structures; in moths and butterflies, the two cups are fused, sharing one part of the wall, whereas in locust and beetles, the structure is formed by a single cup (Brandt et al., 2005; Heinze and Reppert, 2012; Chen et al., 2016; Immonen et al., 2017; von Hadeln et al., 2018). In *A. lucorum*, the calyx is composed of two fused cups with no shared wall. The pedunculus of *A. lucorum* consists of two strands, different from that of locust, fruit fly, beetles, and honeybee, which includes several strands (Brandt et al., 2005; Ito et al., 2014; Immonen et al., 2017; von Hadeln et al., 2018).

The vertical lobe in *A. lucorum* includes only one single component (one α lobe), which differs from that of locust, beetles, fruit fly, moths, and butterflies, consisting of two to three lobelets, e.g., α, α′, and/or γ lobelets (Heinze and Reppert, 2012; Ito et al., 2014; Chen et al., 2016; Immonen et al., 2017; von Hadeln et al., 2018). The medial lobe of *A. lucorum* consists of five β lobelets and two γ lobelets, whereas that of locust, beetles, fruit fly, moths, and butterflies consists of three lobelets, β, β′, and γ. Generally, the organization of the mushroom body lobes in the mirid bug appears relatively complicated. Different from many insects having an accessory calyx, such as the locust, fruit fly, moths, and butterflies, no additional structure associated with the calyx was found in *A. lucorum*.

### Relative Size of Prominent Neuropils Among Different Species

Similar approaches of immunostaining and 3D reconstruction provide anatomical data that can be utilized to compare corresponding neuropils, i.e., the antennal lobes, the optic lobes, the CB, and the mushroom bodies. Thus, in addition to the findings from the mirid bug, present here, anatomical data from 14 different species, i.e., a leafhopper, a planthopper, a locust, a cockroach, a honeybee, three butterflies, three moths, and three ants, were collected to make comparisons (Supplementary Table S3). Since the different species vary in body size from a millimeter to several centimeters, the data were normalized by relating the real size of the neuropils to the size of the whole brain. Overall, it seems that the relative sizes of corresponding neuropils are quite similar within distinct insect taxa. *A. lucorum* possesses the relatively largest antennal lobes and CB, while the optic lobes and mushroom bodies are relatively small (Figure 8). The large antennal lobe and CB in the *A. lucorum* brain might be in line with its biology. The mirid bug is a polyphagous insect that relies heavily on olfaction for selecting host plants and finding mates (Chen et al., 2010; Zhang, 2011; Lu and Wu, 2012; Pan et al., 2015a). Former studies, utilizing a flight-mill system and long-term field observations on an isolated island, showed that *A. lucorum* has high flight potential and can perform even long-distance migration overseas (Lu et al., 2007; Fu et al., 2014). Such migration behaviors are previously shown to be controlled by neurons connected with the CB (Pfeiffer and Homberg, 2014). The relatively large CB in the mirid bug may, therefore, incorporate special networks underlying high motility and complicated patterns of locomotion.

The relatively small size of the optic lobes in *A. lucorum* indicates that visual sensation may not play an eminent role in its life. Interestingly, however, previous behavioral studies have shown that adding light to attractive floral volatiles enhances the attraction of *A. lucorum* significantly (Pan et al., 2015b). This report demonstrates that vision plays an important role in host plant selection. In addition, detection of light is probably critical for ending the diapause of *A. lucorum* eggs and surviving during the cold winter (Feng et al., 2012).

The proportional size of the mushroom bodies is smaller in *A. lucorum* than that in other species. In particular, the calyx of *A. lucorum* is notably small. This structure is innervated by antennal lobe projection neurons, and it is generally assumed that the volume of the calyx is positively correlated to the antennal lobe volume (Strausfeld et al., 2009). In most insects, the calyx receives extensive input from antennal lobe projection neurons. Several species with prominent antennae possess a well-developed antennal lobe and calyx, such as bees, ants, and moths, while species with less developed antennae possess no or weakly developed antennal lobe and calyx, such as dragonflies, cicada, and aquatic insects (Strausfeld et al., 2009). In contrast, *A. lucorum* possesses a large antennal lobe neuropil and a small calyx. Such a unique arrangement of a relatively large antennal lobe and small calyx requires investigation of the neuronal connections and neural circuits rather size measurements.

Actually, the issue implying size should be commented on. Generally, allometry has been used to study facets of an organism’s morphology and physiology in relation to its body size. In a publication from 2004, Smith and Bhatnagar (2004) discussed whether size matters in connection with definition of “microsmatic” and “macrosmatic” organisms. These terms have been utilized to characterize mammals with lesser or greater levels, respectively, of olfactory function. In their publication, Smith and Bhatnagar (2004) plotted olfactory bulb volume vs. body mass as well as total brain volume in different primate taxa. Notably, the authors found that the relationship of olfactory bulb volume to total brain volume was more positive than the relationship between olfactory bulb volume and body mass. They conclude that, for general comparisons, data on olfactory elements from various species should not be scaled to body size. When it comes to relationship of the size of olfactory structures to overall brain size, they say that this requires further exploration. Among the various challenges listed up by the authors is the architecture of the olfactory bulb including a six-layered tissue surrounding a non-neuronal fluid-filled cavity showing marked variation between different taxa. Even though the insect antennal lobe displays a more uniform arrangement across different species, we should keep in mind that proportionately large antennal lobes may not necessarily mean an improved ability of odor discrimination.

## Conclusion

In summary, *A. lucorum* has a general brain composition similar to that of other insect species, but with substantial expansion of distinct regions, such as the antennal lobes and the CB. To our surprise, the antennal lobe of the small bug, *A. lucorum*, is extremely large and its relative size (meaning the size relative to the whole brain volume) is the biggest among all insect species studied so far. Even though expansion of distinct brain structures often indicates increased investment in functionally important neuropils, further studies should focus on exploring the neural circuit forming these neuropils. The knowledge about the brain structure of *A. lucorum* will facilitate studies on the neural basis of its complicated behaviors. Here, studies on the neural arrangements involved in olfaction will be highly relevant.

## Data Availability

All datasets generated for this study are included in the manuscript and/or the Supplementary Files.

## Author Contributions

G-YX and X-CZ contributed to the study concept and design. G-YX, B-WM, X-LL, Y-JC, W-BC, and G-PL contributed to the acquisition of data. G-YX, X-CZ, H-QF, and Y-JZ contributed to the analysis and interpretation of data. X-CZ and BB contributed to the drafting of the manuscript. G-YX and X-CZ obtained the funding. All authors read and approved the final manuscript.

## Conflict of Interest Statement

The authors declare that the research was conducted in the absence of any commercial or financial relationships that could be construed as a potential conflict of interest.
