# Supplementary material for: Brain Organization of Apolygus lucorum: A Hemipteran Species With Prominent Antennal Lobes
Source: Front Neuroanat. 2019 Jul 17;13:70. doi: 10.3389/fnana.2019.00070 (PMC6654032; doi:10.3389/fnana.2019.00070)
Supplement: Supplementary file 1 [file Table_1.doc]

**Table S1** Absolute volumes and relative sizes of brain neuropils in *Apolygus lucorum*

|  | Male | | | Female | | |
| --- | --- | --- | --- | --- | --- | --- |
| Item | n | Volume (10^4^ μm³)  mean±SD | Relative to brain% mean ±SD | n | Volume (10^4^ μm³)  mean±SD | Relative to brain% mean ±SD |
| R-LA | 6 | 33.25±11.81 | 4.19±0.72 | 4 | 26.57±5.93 | 3.55±0.49 |
| L-LA | 6 | 32.46±14.18 | 3.98±1.11 | 4 | 24.56±5.11 | 3.42±0.35 |
| R-ME | 6 | 40.95±12.69 | 5.22±0.40 | 4 | 42.76±2.48 | 5.30±0.31 |
| L-ME | 6 | 42.73±17.49 | 5.249±1.05 | 4 | 47.34±3.24 | 6.13±0.87 |
| R-LOX | 6 | 13.68±3.85 | 1.75±0.11 | 4 | 14.14±0.44 | 1.77±0.16 |
| L-LOX | 6 | 12.86±4.62 | 1.61±0.28 | 4 | 14.22±2.43 | 1.91±0.14 |
| R-AOTU | 6 | 1.92±0.58 | 0.25±0.05 | 5 | 1.57±0.32 | 0.27±0.03 |
| L-AOTU | 6 | 1.68±0.70 | 0.22±0.06 | 5 | 1.43±0.41 | 0.21±0.07 |
| R-CA | 6 | 3.93±1.42 | 0.51±0.11 | 5 | 4.39±0.83 | 0.54±0.13 |
| L-CA | 6 | 4.04±1.78 | 0.51±0.15 | 5 | 4.49±0.84 | 0.50±0.08 |
| R-PED | 6 | 2.09±0.65 | 0.27±0.05 | 4 | 2.65±0.53 | 0.33±0.06 |
| L-PED | 6 | 2.06±0.83 | 0.26±0.05 | 4 | 2.61±0.63 | 0.35±0.06 |
| R-LOB | 6 | 10.26±3.09 | 1.33±0.28 | 4 | 12.12±3.02 | 1.65±0.29 |
| L-LOB | 6 | 10.86±3.29 | 1.40±0.22 | 4 | 12.37±1.75 | 1.60±0.15 |
| CB | 6 | 8.52±2.33 | 1.11±0.15 | 4 | 9.93±1.41 | 1.29±0.24 |
| R-PB | 6 | 1.38±0.66 | 0.17±0.05 | 4 | 1.06±0.39 | 0.16±0.04 |
| L-PB | 6 | 1.26±0.66 | 0.16±0.06 | 4 | 1.12±0.17 | 0.15±0.01 |
| R-LAL | 6 | 6.38±3.52 | 0.85±0.47 | 4 | 6.87±2.84 | 1.03±0.31 |
| L-LAL | 6 | 6.07±1.83 | 0.83±0.39 | 4 | 5.78±3.11 | 0.85±0.38 |
| R-AL | 6 | 57.58±24.99 | 7.12±1.56 | 5 | 55.16±4.71 | 7.12±0.50 |
| L-AL | 6 | 57.60±20.86 | 7.25±0.95 | 5 | 52.69±9.09 | 7.56±1.43 |
| R-TR | 6 | 7.53±2.98 | 0.96±0.27 | 5 | 8.30±2.00 | 1.02±0.24 |
| L-TR | 6 | 7.37±2.88 | 0.94±0.22 | 5 | 8.57±1.66 | 1.07±0.19 |
| GNG | 6 | 108.24±20.32 | 14.34±2.14 | 5 | 99.67±20.62 | 13.32±2.99 |
| midbrain | 6 | 305.20±77.85 | 39.46±1.87 | 5 | 322.91±48.98 | 38.94±2.17 |
| SUM brain | 6 | 779.89±219.56 | - | 3 | 788.94±67.14 | - |

Midbrain: the remaning neuropils of in the central brain except those prominent neuropils, MB, CX, LAL, and AOTU. ”-” not calculated.

R, right; L, left. AL, antennal lobe; AOTU, anterior optic tubercle; CA, calyx; CB, central body; LA, lamina; LAL, lateral accessory lobe; LOB, mushroom-body lobes; LOX, lobula complex; ME, medulla; PB: protocerebral bridge; PED, pedunculus; TR, tritocerebrum.
