# Supplementary material for: Brain Organization of Apolygus lucorum: A Hemipteran Species With Prominent Antennal Lobes
Source: Front Neuroanat. 2019 Jul 17;13:70. doi: 10.3389/fnana.2019.00070 (PMC6654032; doi:10.3389/fnana.2019.00070)
Supplement: Supplementary file 2 [file Table_2.doc]

**Table S2** Mann-Whitney U test for differences in size of the neuropils between the right and left hemispheres, and between male and female

| Item | Right and left neuropils in male | | Right and left neuropils in female | | Male and  female |
| --- | --- | --- | --- | --- | --- |
|  | n | *P* | n | *P* | *P* |
| LA | 6 | 0.818 | 4 | 1.000 | 0.257 |
| ME | 6 | 0.699 | 4 | 0.057 | 1.000 |
| LOX | 6 | 0.699 | 4 | 0.486 | 0.476 |
| AOTU | 6 | 0.485 | 5 | 0.548 | 0.537 |
| CA | 6 | 1.000 | 5 | 1.000 | 0.429 |
| PED | 6 | 1.000 | 4 | 0.686 | 0.257 |
| LOB | 6 | 0.818 | 4 | 0.886 | 0.610 |
| CB | 6 | - | 4 | - | 0.352 |
| PB | 6 | 0.699 | 4 | 0.686 | 0.914 |
| LAL | 6 | 0.818 | 4 | 1.000 | 1.000 |
| AL | 6 | 0.699 | 5 | 0.548 | 0.537 |
| TR | 6 | 1.000 | 5 | 1.000 | 0.537 |
| GNG | 6 | - | 5 | - | 0.662 |
| midbrain | 6 | - | 5 | - | 0.931 |
| SUM brain | 6 | - | 3 | - | 0.548 |

Midbrain: the remaining neuropils of in the central brain except for the MB, CX, LAL, and AOTU. “-” not calculated.

AL, antennal lobe; AOTU, anterior optic tubercle; CA, calyx; CB, central body; GNG, gnathal ganglion; LA, lamina; LAL, lateral accessory lobe; LOB, mushroom-body lobes; LOX, lobula complex; ME, medulla; PB: protocerebral bridge; PED, pedunculus; TR, tritocerebrum.
