# Supplementary material for: Brain Organization of Apolygus lucorum: A Hemipteran Species With Prominent Antennal Lobes
Source: Front Neuroanat. 2019 Jul 17;13:70. doi: 10.3389/fnana.2019.00070 (PMC6654032; doi:10.3389/fnana.2019.00070)
Supplement: Supplementary file 3 [file Table_3.doc]

**Table S3** Absolute volumes of selected neuropils in some studied insect species

| Species | Volume (10^4^ μm³) | | | | | | | References |
| --- | --- | --- | --- | --- | --- | --- | --- | --- |
|  | AL | OL (ME+LOX) | CB | MB | CA | LOB(PED+LOB)^1^ | Sum of brain |  |
| Blattodea |  |  |  |  |  |  |  |  |
| *Diploptera punctata* | 440.00 | - | 350.00 | 2660.00 | 900.00 | 1760.00 | 50930.00 | Chiang et al., 2003 |
| Orthoptera |  |  |  |  |  |  |  |  |
| *Schistocerca gregaria* | 3830.00 | 39064.00 | 664.70 | 6086.00 | 3556.00 | 2530.00 | 69669.36 | Kurylas et al., 2008 |
| Hemiptera |  |  |  |  |  |  |  |  |
| *Apolygus lucorum* | 112.87 | 114.37 | 9.08 | 35.53 | 8.47 | 27.06 | 782.91 | this study |
| *Scaphoideus titanus* | 2.28 | - |  | - |  |  | 2439.50 | Rossi Stacconi et al., 2014 |
| *Hyalesthes obsoletus* | 21.99 | - |  | - |  |  | 3871.50 | Rossi Stacconi et al., 2014 |
| Lepidoptera |  |  |  |  |  |  |  |  |
| *Heliothis virescens*^2^ | 865.00 | 3556.00 | 169.00 | 751.00 | 476.00 | 275.00 | 14750.80 | Kvello et al., 2009 |
| *Helicoverpa assulta*^2^ | 880.00 | 4902.00 | 132.00 | 810.00 | 494.00 | 316.00 | 15187.00 | Chen et al., 2016 |
| *Manduca sexta* | 6780.00 | 38480.00 | 441.50 | 3325.00 | 1858.50 | 1466.50 | 102012.76 | el Jundi et al., 2009 |
| *Godyris zavaleta* | 854.00 | 8294.00 | 81.00 | 395.90 | 240.00 | 155.90 | 17120.00 | Montogomery and Ott, 2015 |
| *Heliconius erato* | 1185.00 | 27983.00 | 148.17 | 7543.30 | 4672.00 | 2871.30 | 64174.27 | Montogomery et al., 2016 |
| *Heliconius hecale* | 1216.00 | 34564.00 | 140.39 | 8360.00 | 5271.00 | 3089.00 | 79953.54 | Montogomery et al., 2016 |
| Hymenoptera |  |  |  |  |  |  |  |  |
| *Apis mellifera* | 2170.00 | 14740.00 | 232.00 | 8312.00 | 5162.00 | 3150.00 | 38154.00 | Brandt et al., 2005 |
| *Camponotus sericeus* | 707.50 | 617.50 |  | 1700.00 |  |  | 8750.00 | Mysore et al., 2009 |
| *Camponotus compressus* | 1000.00 | 512.00 |  | 2260.00 |  |  | 11000.00 | Mysore et al., 2009 |
| *Camponotus floridanus* | 296.27 | - |  | - |  |  | 2581.74 | Zube et al., 2008 |

“-” not calculated.

^1^The PED of *Apolygus lucorum* was included in LOB, which was included in LOB of other species in the reports listed here.

^2^The AL volumes of *Heliothis virescens* and *Helicoverpa assulta*, measured without the core of AL originally, were corrected to estimate total AL volume based upon the description of Montgomery and Ott (2015). AL, antennal lobe; CA, calyx; CB, central body; LOB, mushroom-body lobes; LOX, lobula complex; MB, mushroom body; ME, medulla; OL, optic lobe; PED, pedunculus.

**References**

Brandt, R., Rohlfing, T., Rybak, J., Krofczik, S., Maye, A., Westerhoff, M., Hege, H.C., and Menzel, R. (2005). Three-dimensional average-shape atlas of the honeybee brain and its applications. *J. Comp. Neurol.* 492(1), 1-19.

Chen, Q.Y., Wu, X., Tang, Q.B., Xie, G.Y., and Zhao, X.C. (2016). Anatomical organization and three-dimensional reconstruction of the brain in adult *Helicoverpa assulta* (Lepidoptera: Noctuidae). *Acta Entomol. Sin.* 59(1), 33-46.

Chiang, A.S., Liu, Y.C., Chiu, S.L., Hu, S.H., Huang, C.Y., and Hsieh, C.H. (2001). Three-deimensional mapping of brain neuropil in the cockroach *Diploptera punctate*. *J. Comp. Neurol.* 440, 1-11.

el Jundi, B., Huetteroth, W., Kurylas, A.E., and Schachtner, J. (2009). Anisometric brain dimorphism revisited: implementation of a volumetric 3D standard brain in *Manduca sexta*. *J. Comp. Neurol.* 517, 210-225.

Kurylas, A.E., Rohlfing, T., Krofczik, S., Jenett, A., and Homberg, U. (2008). Standardized atlas of the brain of the desert locust, *Schistocerca gregaria*. *Cell Tissue Res.* 333, 125-145.

Kvello, P., Løfaldli, B.B., Rybak, J., Menzel, R., and Mustaparta, H. (2009). Digital, three-dimensional average shaped atlas of the *Heliothis virescens* brain with integrated gustatory and olfactory neurons. *Front. Syst. Neurosci.* 3,14.

Montogomery, S.H., and Ott, S.R. (2015). Brain composition in *Godyris zavaleta*, a diurnal butterfly, reflects an increased reliance on olfactory information. *J. Comp. Neurol.* 523, 869-891.

Montogomery, S.H., Merrill, R.M., and Ott, S.R. (2016). Brain composition in *Heliconius* butterflies, postecolosion growth and experience-dependent neuropil plasticity. *J. Comp. Neurol.* 524, 1747-1769.

Mysore, K., Subramanian, K.A., Sarasij, R.C., Suresh, A., Shyamala, B.V., VijayRaghava,n K., and Rodrigues, V. (2009). Caste and sex specific olfactory glomerular organization and brain architecture in two sympatric ant species *Camponotus sericeus* and *Camponotus compressus* (Fabricius, 1798). *Arthropod Struct. Dev.* 38, 485-497.

Rossi Stacconi, M.V., Hansson, B.S., Rybak, J., and Romani, R. (2014). Comparative neuroanatomy of the antennal lobe of 2 homopteran species. Chem. Senses 39, 283-294.

Zube, C., and Rössler, W. (2008). Caste- and sex-specific adaptations within the olfactory pathway in the brain of the ant *Camponotus floridanus*. *Arthropod Struct. Dev.* 37, 469-479.
